# The efficacy of treadmill training with and without projected visual context for improving walking ability and reducing fall incidence and fear of falling in older adults with fall-related hip fracture: a randomized controlled trial

**DOI:** 10.1186/s12877-016-0388-x

**Published:** 2016-12-28

**Authors:** Mariëlle W. van Ooijen, Melvyn Roerdink, Marga Trekop, Thomas W. J. Janssen, Peter J. Beek

**Affiliations:** 1 Department of Human Movement Sciences, Faculty of Behavioural and Movement Sciences, Vrije Universiteit Amsterdam, MOVE Research Institute Amsterdam, Van der Boechorststraat 9, Amsterdam, 1081 BT The Netherlands; 2 Amsterdam Rehabilitation Research Center | Reade, Overtoom 283, Amsterdam, 1054 HW The Netherlands; 3 PW Janssen, Zorggroep Solis, Hermelijn 2, Deventer, 7423 EJ The Netherlands

**Keywords:** Walking adaptability, Falls, Older adults, Hip fracture, Intervention studies, Treadmill, Exercise

## Abstract

**Background:**

The ability to adjust walking to environmental context is often reduced in older adults and, partly as result of this, falls are common in this population. A treadmill with visual context projected on its belt (e.g., obstacles and targets) allows for practicing step adjustments relative to that context, while concurrently exploiting the great amount of walking practice associated with conventional treadmill training. The present study was conducted to compare the efficacy of adaptability treadmill training, conventional treadmill training and usual physical therapy in improving walking ability and reducing fear of falling and fall incidence in older adults during rehabilitation from a fall-related hip fracture.

**Methods:**

In this parallel-group, open randomized controlled trial, seventy older adults with a recent fall-related hip fracture (83.3 ± 6.7 years, mean ± standard deviation) were recruited from inpatient rehabilitation care and block randomized to six weeks inpatient adaptability treadmill training (*n* = 24), conventional treadmill training (*n* = 23) or usual physical therapy (*n* = 23). Group allocation was only blind for assessors. Measures related to walking ability were assessed as the primary outcome before and after the intervention and at 4-week and 12-month follow-up. Secondary outcomes included general health, fear of falling, fall rate and proportion of fallers.

**Results:**

Measures of general walking ability, general health and fear of falling improved significantly over time. Significant differences among the three intervention groups were only found for the Functional Ambulation Category and the dual-task effect on walking speed, which were in favor of respectively conventional treadmill training and adaptability treadmill training.

**Conclusions:**

Overall, adaptability treadmill training, conventional treadmill training and usual physical therapy resulted in similar effects on walking ability, fear of falling and fall incidence in older adults rehabilitating from a fall-related hip fracture. Additional post hoc subgroup analyses, with stratification for pre-fracture tolerated walking distance and executive function, revealed several intervention effects in favor of adaptability and conventional treadmill training, indicating superiority over usual physical therapy for certain subgroups. Future well-powered studies are necessary to univocally identify the characteristics of individuals who will benefit most from a particular intervention.

**Trial registration:**

The Netherlands Trial Register (NTR3222, 3 January 2012).

**Electronic supplementary material:**

The online version of this article (doi:10.1186/s12877-016-0388-x) contains supplementary material, which is available to authorized users.

## Background

Falls are common in older adults and may have major consequences [1–3]. Safe walking requires the ability to make step adjustments in response to environmental demands, an ability that is reduced in older adults [4, 5]. Falls occur most commonly during walking and environmental hazards contribute to approximately half of all falls [2, 3, 6]. Interventions that incorporate overground walking adaptability exercises have shown improved obstacle avoidance performance and reduced fall incidence in older adults [7, 8]. In line with these findings, a recent systematic review and meta-analysis showed that falls in older adults can be reduced by about 50% after stepping training in response to environmental challenges [9]. Practicing the complex and hazardous situations of everyday walking is therefore important to prevent falls [9], but older adults have little opportunity to do so consistently and safely in a task-specific manner.

**Fig. 1:**
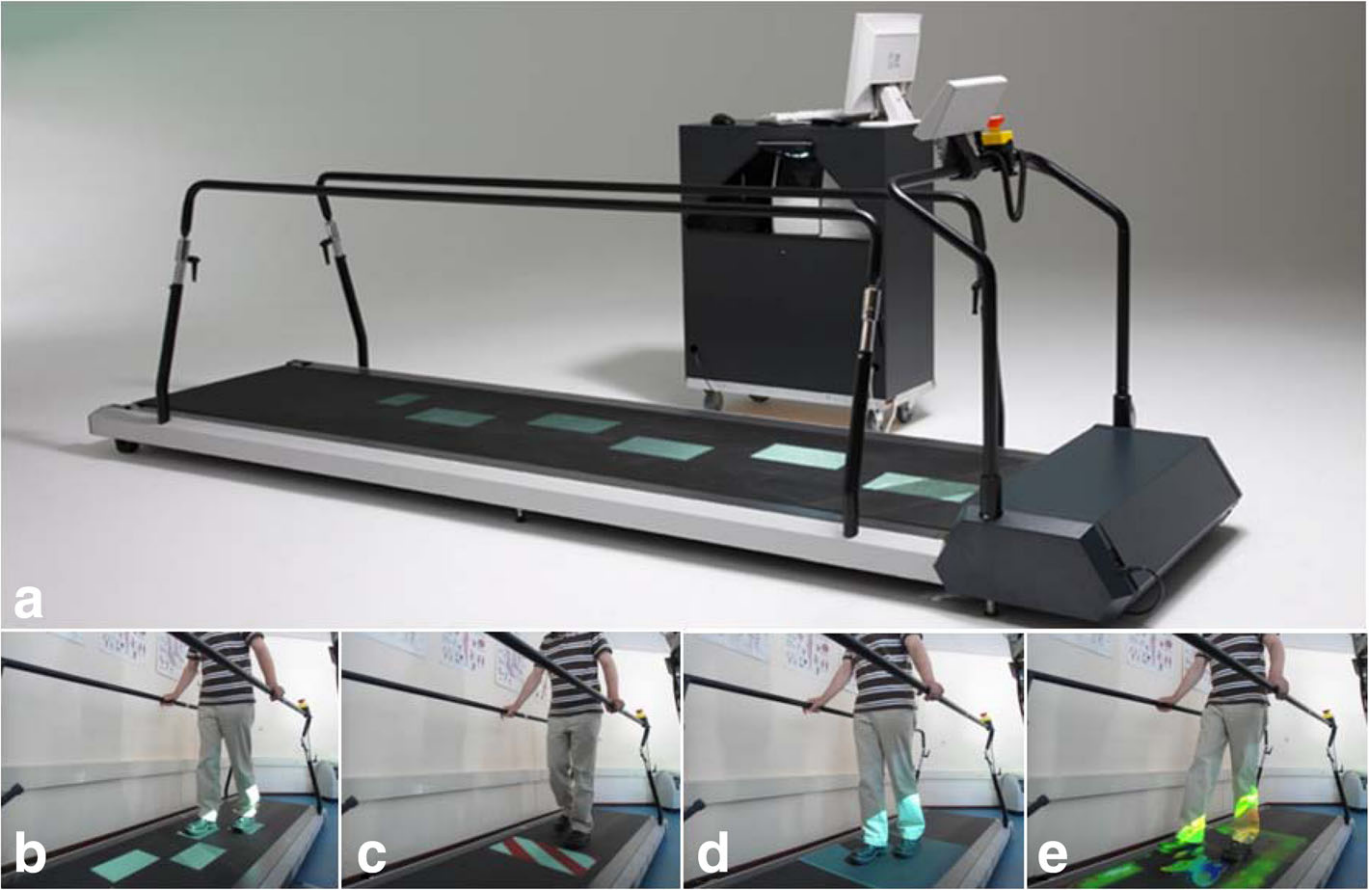
Adaptability treadmill training. The instrumented treadmill with visual context presented on the belt’s surface using a projector. The handrail and an emergency stop allow for a safe practice environment (**a**). The walking adaptability exercises include visually guided stepping to a sequence of regular or irregular stepping targets (**b**), obstacle avoidance (**c**), speeding up and slowing down by following a moving walking zone (**d**) and interactive walking adaptability games (**e**)

The C-Mill (Motekforce Link, Amsterdam/Culemborg, The Netherlands) is an instrumented treadmill for practicing step adjustments during walking. Visual context (e.g., obstacles and targets) is projected on the belt`s surface to elicit task-specific step adjustments during walking (Fig. 1), mimicking the step adjustments required for safe community ambulation in a cluttered environment [10]. Previous research has revealed promising results of this type of training in persons with neurological impairment, in the form of improved balance, walking [11–14] and obstacle avoidance performance [11–13]. In addition, this improved obstacle avoidance was found to be associated with reduced attentional demands of adaptive walking [13].

Van Ooijen et al. [15] recently found that adaptability treadmill training was well received and tolerated by older adults recovering from a fall-related hip fracture. Moreover, adaptability treadmill training and conventional treadmill training led to greater amounts of walking practice than usual physical therapy in older adults with fall-related hip fracture [15]. Although the amount of practice is generally regarded as an important factor for effective rehabilitation [16–18], it has not been examined to date whether the use of a treadmill results in better walking ability and reduced fall incidence in older adults.

The present study compared the efficacy of adaptability treadmill training, conventional treadmill training and usual physical therapy on walking ability, fear of falling and fall incidence in older adults after a fall-related hip fracture, with the aim to establish the usefulness of treadmill walking with and without walking adaptability exercises in fall prevention programs [19]. We expected better outcomes related to general walking ability after adaptability and conventional treadmill training than after usual physical therapy due to the greater amounts of walking practice. We further expected superior task-specific effects for adaptability treadmill training on adaptability aspects of walking, fear of falling and fall incidence in view of its concurrent focus on practicing walking adaptability.

## Methods

This parallel group, single-blind, superiority randomized controlled trial was registered in the Netherlands Trial Register (NTR3222) and approved by the Medical Ethical Reviewing Committee of VU University Medical Centre, Amsterdam, The Netherlands. The trial was previously described in detail [19].

### Participants

We aimed to recruit 126 participants with a fall-related hip fracture from residential and rehabilitation center Zorggroep Solis in Deventer, The Netherlands. After discharge from the hospital, most patients recovering from a hip fracture are referred to a residential and rehabilitation centre when they need additional temporary care before returning to their homes. All patients with a hip fracture discharged from a hospital and admitted to residential and rehabilitation centre Zorggroep Solis, Deventer, The Netherlands, were assessed for participation eligibility within 3 days from admission by a physical therapist during a regular intake session. Inclusion criteria were admission with a hip fracture related to falling, ≥ 65 years of age, Functional Ambulation Category score 2 or higher (FAC [20, 21]), expected duration of admission ≥ 6 weeks and an ability to understand and execute simple instructions. Exclusion criteria were: not being allowed to bear weight on the affected leg, moderate or severe cognitive impairments as indicated with a score below 18 at the Mini-Mental State Examination (MMSE [22]), severe non-corrected visual impairments limiting the correct perception of the direct environment, contraindication to physical activity and an activity tolerance below 40 min with rest intervals. Patients eligible for participation were informed of the study by the physical therapist, both verbally and in writing. All included participants gave written informed consent. Data analyses included all participants who had completed at least 4 weeks of intervention.

### Study design

After completing pre-intervention assessments (T0), participants were block randomized to six weeks of inpatient adaptability treadmill (AT) training, conventional treadmill (CT) training or usual physical therapy (UPT). The six-week intervention period comprised 30 training sessions (i.e., five sessions per week) of 40 min each. Training sessions were conducted by physical therapists, typically with two participants supervised by one physical therapist. Participants alternately practiced and rested during the 40-min training sessions, resulting in 20 min of actual practice for each participant. Inevitably, physical therapists and participants were not blinded to group allocation. However, post-intervention assessments (T1) and follow-up assessments at four weeks (T2) and 12 months (T3) after the intervention period were conducted by an independent assessor, who was blinded to group allocation. Falls were monitored monthly between T1 and T3 [19].

### Intervention

Participants in the UPT group received 30 sessions of conventional physical therapy, including exercises of leg strength (e.g., hip abduction, flexion and extension, knee extension and ankle dorsi-plantar flexion when lying, sitting or standing), balance (e.g., stance), transfers (e.g., bed to chair, chair to toilet, chair to chair, sit to stance and vice versa), overground walking (e.g., short bouts of unconstrained walking between parallel bars, in the practice hall or outside, walking backwards, walking sideways and walking an obstacle course) and activities of daily living (e.g., climbing stairs, bringing cups to the kitchen, putting cups in the closet, and opening and closing the curtains). These training sessions followed locally implemented guidelines regarding the treatment of hip fractures and aimed to facilitate the participant’s return to home. Participants used their walking aid during the sessions of usual physical therapy and the physical therapist provided verbal instructions or occasional physical assistance when necessary.

For the *conventional treadmill* (CT) training group, 15 of the 30 UPT sessions were replaced by treadmill walking. The physical therapist and the participant jointly determined the treadmill walking speed to promote the quality and safety of walking at a speed that was reported as comfortable by the participant. The focus of conventional treadmill training sessions was initially on the quality and safety aspects of walking and gradually shifted towards walking faster and longer. Participants walked on the C-Mill treadmill without projection of visual context and used no body weight support other than the handrail.

The *adaptability treadmill* (AT) training group performed 15 sessions of usual physical therapy and 15 sessions of adaptability treadmill training. As in CT training, participants walked at a comfortable walking speed and without bodyweight support other than using the handrail. The first two AT training sessions consisted of conventional treadmill training to become acquainted with treadmill walking. Subsequent AT training sessions were specifically focused on practicing walking adjustments in response to the visual context projected on the C-Mill (Fig. 1). C-Mill walking adaptability exercises consisted of visually guided stepping to a sequence of regularly or irregularly spaced stepping targets (Fig. 1b) with or without targets changing to obstacles, obstacle avoidance (Fig. 1c), speeding up and slowing down by following a projected walking area oscillating in anterior-posterior direction over the treadmill surface (Fig. 1d) and walking adaptability games consisting of interactive stepping targets (e.g., beach balls) and obstacles (e.g., seals, shells, crabs) (Fig. 1e). Further details on the interventions can be found in van Ooijen et al. [19].

### Outcome measures and data analysis

#### Demographic and clinical characteristics

Demographics, medical information and pre-fracture functioning were obtained from medical files, including comorbidities, medication use, type of hip fracture, type of surgery and pre-fracture FAC score, living situation, ambulatory assistance and tolerated walking distance. Cognitive function at baseline was measured using the Mini-Mental State Examination (MMSE [22]).

#### Walking ability: general walking ability and walking adaptability

Measures related to walking ability were the primary outcomes of the present study. General walking ability was assessed using a complementary set of standard clinical tests related to mobility, walking and daily functioning. We performed the performance oriented mobility scale (POMA [23, 24]), Elderly Mobility Scale (EMS [25]) and Timed Up-and-Go test (TUG [26]) as measures of mobility, covering walking, balance and positional changes. The Functional Ambulation Category (FAC [20, 21]) and the 10 m Walk Test (10MWT [27]) were performed to assess independence of walking and walking speed, respectively. The Nottingham Extended Activities of Daily Living scale (NEADL [28]) was administered to evaluate activities of daily living.

Walking adaptability was assessed with two more complex walking tasks, by modifying the standard 10MWT with three obstacles in the walkway and by asking participants to concurrently subtract 3’s from a random number between 191 and 199. This allowed us to assess obstacle avoidance performance and the dual-task effects on walking speed, respectively [29]. Obstacle avoidance performance was operationalized using the definition of dual-task effect: obstacle effect = (walking speed with obstacles - walking speed without obstacle)/walking speed without obstacles * 100% [30]. In addition, the percentage of successfully avoided obstacles was calculated. The serial-3 subtraction task was also performed while being seated; the performance on this task was defined as the number of correct subtractions scaled to 60 s. Dual-task walking was assessed by calculating the dual-task effects (DTE) on the walking task as well as on the serial-3 subtraction task: (dual-task performance - single-task performance)/single-task performance * 100% [30]. As such, negative (positive) DTE values indicate that performance declined (improved) in the dual task compared with the single task.

The 10MWTs and TUG test were performed twice, with the average performance of both trials used as outcome. The 10MWTs and TUG were only performed immediately after the intervention period and four weeks later because these tests were too onerous prior to the intervention and too difficult to assess during the home visits at 12-month follow-up. An overview of the tests performed at each measurement including a more detailed description of the outcome measures is presented in Table 1.

**Table 1:** Study assessment schedule

		T0	T1	T2	T3
Primary outcome measure related to general walking ability					
Performance Oriented Mobility Assessment (POMA)	Widely used tool for assessing mobility and fall risk in older people. The assessment examines several qualitative aspects of the locomotion pattern and carries the subject through positions and changes in positions, reflecting stability tasks that are related to daily activities. Each item is scored on a 2- or 3-point scale (0–1 or 0-1-2 points), resulting in a maximum score of 28 [24].	x	x	x	x
Elderly Mobility Scale (EMS)	Covers locomotion, balance and key position changes which are prerequisites to more complex activities of daily living by testing: lying to sitting, sitting to lying, sitting to standing, standing, gait, walking speed and functional reach. The maximum score possible, which represents independent mobility is 20, the minimum score is 0 [25].	x	x	x	x
Timed Up-and-Go test (TUG)	A measure of functional mobility. The participant is asked to rise from a standard chair, walk to a line on the floor 3 m away, turn, return and sit down again. The score given is the time taken in seconds to complete the test [26].		x	x	
Functional Ambulation Category (FAC)	A quick visual measurement of the independence of walking. The FAC distinguishes 6 levels of walking ability based on the amount of human assistance required [20, 21].	x	x	x	x
10 m Walking Test (10MWT)	Measures the walking speed over 10 m [27].		x	x	
Nottingham Extended Activities of Daily Living (NEADL)	Measures the performance of activities of daily living. The NEADL comprises 22 items divided into four sections: mobility, kitchen, domestic and leisure. Each item is given one of four responses (able 3 pt, able with difficulty 2 pt, able with help 1 pt, unable 0 pt) [28].	x		x	x
Primary outcome measures related to walking adaptability					
10 m Walking Test with obstacles (10MWT_obstacle_)	This is a 10 m walk test with 3 obstacles in the walkway to evaluate obstacle avoidance during walking. Two obstacles of 5×20×10 cm [height× width× length] are placed at 2.5 and 7.5 m from the starting line and one obstacle of 10×20×5 cm is placed at 5 m from the starting line.		x	x	
10 m Walking Test with cognitive task (10MWT_cognitive_)	This is a 10 m walk test that is performed while participants subtract 3’s from a random number between 191 and 199. This test is performed to evaluate the cognitive dual-task effect of walking.		x	x	
Secondary outcome measure, related to fear of falling					
Falls Efficacy Scale International (FES-I)	Measures confidence in performing a range of specific activities of daily living without falling. The scale consists of 20 items of which each is responded with one of the following answers: not at all concerned (1 pt), somewhat concerned (2 pt), fairly concerned (3 pt), very concerned (4 pt) [31].	x	x	x	x
Secondary outcome measure, related to fall incidence					
Monthly fall diary	Falls and near falls are monitored monthly from T1 to T3 using a daily calendar diary for postal use [35].				x
Secondary outcome measures, related to general health					
Visual Analogue Scale of perceived general health (VAS)	Participants rate their presently perceived general health using a visual analogue scale (VAS) ranging from 0 (worst imaginable health state) to 100 (best imaginable health state).	x	x	x	x
Hip Disability and Osteoarthritis Score - Quality of life (HOOS-Q)	This subscale of the HOOS measures hip-related quality of life and consists of 4 questions. Standardized response options are given (5-point Likert scale) and each of the 4 questions is scored from 0 to 4; then a normalized score (100 indicating no symptoms and 0 indicating extreme symptoms) is calculated for the subscale [32].			x	x
Trail Making Test – parts A and B (TMTa, TMTb)	The TMT was performed to evaluate executive function, which comprises multiple cognitive processes (i.e., visual scanning, task shifting, planning and mental flexibility). Part A of the TMT consists of 25 circles distributed over an entire page and numbered 1 to 25. The participant is required to connect the circles with a pencil line as quickly as possible, beginning with 1 and proceeding in numerical sequence. Part B consists of 25 circles, numbered 1 to 13 and lettered from A to L. The participant is required to connect the circles, but alternating between numbers and letters and taking both series in ascending sequence. The score for the test was the number of seconds required for completion of each part [33].	x	x	x	x

#### Fear of falling and general health

The Falls Efficacy Scale International (FES-I [31]) was performed as a measure of perceived fear of falling. Perceived general health was assessed using a visual analogue scale (VAS) ranging from 0 (worst imaginable health state) to 100 (best imaginable health state). The subscale Quality of life of the Hip disability and Osteoarthritis Score (HOOS-Q [32]) was administered to assess self-perceived limitations related to the hip. The Trail Making Test (parts A and B; TMTa, TMTb [33]) was performed to evaluate executive function, which comprises multiple cognitive processes (i.e., visual scanning, task shifting, planning and mental flexibility) and has been associated with gait impairments, reduced obstacle avoidance ability and falling [29, 34].

#### Falls

Falls and near falls were monitored monthly from T1 to T3 using a daily calendar diary for postal use [35]. Falls were defined as ‘unintentionally coming to rest on the ground or other lower level’, while near falls were defined as ‘a loss of balance that causes an experience of starting to fall which was eventually prevented’ [1, 8, 36]. Circumstances of falls and near falls were reported on the daily calendar diary to check whether the reported (near) falls met the definition of a (near) fall. If a calendar was not returned or information was incomplete, participants were reminded to return the calendar; alternatively, the missing information was obtained by phone. The number of falls and the number (proportion) of fallers (persons with at least one fall) in each group were used as outcome measures. Fall data analyses included the participants who registered falls for at least 6 months.

### Statistical analysis

The sample size was based on previous clinical trials using the POMA as a primary outcome measure [37, 38]. Using a between-group mean difference of 3.5 POMA points, a standard deviation of 4.8 POMA points and a correlation coefficient of 0.7, sample size calculations revealed that 126 participants were required to achieve a statistical power of 80% with a two-tailed conservative alpha of 0.017 corrected for multiple comparisons and an expected drop-out rate of 24% [19].

Participant characteristics and baseline performance were compared among the three groups using One-Way ANOVAs for normally distributed interval variables, Kruskal-Wallis tests for ordinal and non-normal interval variables and Fisher’s exact tests for nominal variables. Analysis of covariance (ANCOVA) with the baseline value of the dependent variable as covariate was used to compare the effect of the intervention among groups at T1, T2 and T3. Outcome measures not registered at baseline were compared among groups at T1, T2 and T3 using One-Way ANOVAs for normally distributed variables and Kruskal-Wallis tests for non-normally distributed variables. The assumption of normality was checked by visually inspecting the data within groups or the standardized residuals (for ANCOVA) using QQ-plots and boxplots. For ordinal variables (FAC), we used Kruskal-Wallis tests to compare the change scores relative to baseline at T1, T2 and T3 among groups. Significant effects were followed by least significant difference (LSD) post hoc tests for normally distributed variables and Mann–Whitney *U* tests for ordinal and non-normally distributed variables.

Since we were also interested in the effect of time on the outcome measures, we performed mixed Time × Intervention repeated measures ANOVAs as secondary analyses. Given the considerable number of dropouts from T2 to T3, we performed these mixed repeated measures ANOVAs both with and without the T3 measurement. Only the results of the mixed repeated measures ANOVAs without the T3 measurement are reported, because results of the analyses with and without T3 measurements were similar and several outcomes were not reported at T3. In addition, those outcomes reported at T3 were reported in much fewer participants than at T2. For ordinal and non-normal interval variables, the effect of time was evaluated using Friedman tests and/or Wilcoxon signed rank tests. For non-normal variables registered at T1 and T2 only, Kruskal-Wallis tests were used to compare the change from T1 to T2 among groups.

Fall incidence rates were calculated for each group and were compared among groups using negative binomial regression analysis adjusted for observation time. Results were reported using fall incidence rate ratios with their 95% confidence interval (CI). The proportion of fallers in each group was compared using relative risks with 95% CIs.

All statistical tests were two-tailed and performed in SPSS 21 (SPSS Inc, IBM Corporation, New York, USA). The level of significance was set at *p* < 0.05 for the main analyses and at *p* < 0.01 for post hoc tests to correct for multiple comparisons. Results are reported as frequency (proportion) for nominal data, as median (minimum-maximum) for ordinal or non-normal interval data and as mean ± standard deviation for normally distributed interval and ratio data. Effect sizes are presented as partial eta squared (*η*_*p*_^*2*^) for ANCOVA and One-Way ANOVA, and as *r* for Wilcoxon signed rank tests. Effect sizes for Kruskal-Wallis tests were not reported, because straight effect size measures are not available for this test. However, effect sizes of the associated post hoc Mann-Whitney *U* tests are reported as *r*, which also applies for LSD post hoc tests [39].

## Results

**Fig. 2:**
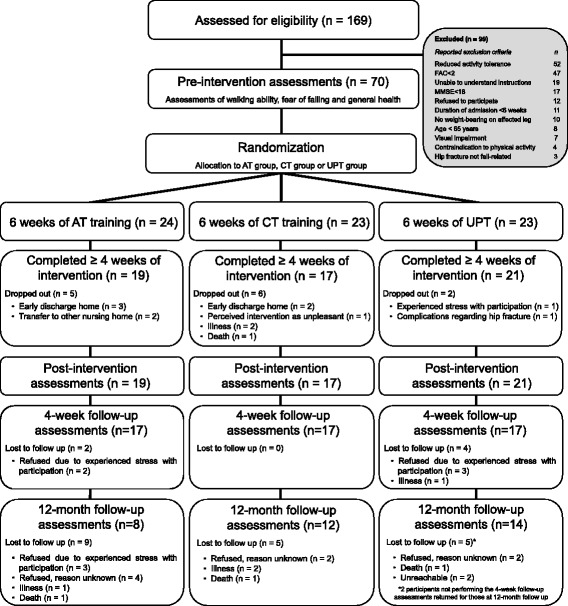
Flow chart of the study procedures. Abbreviations: AT: adaptability treadmill, CT: conventional treadmill, UPT: usual physical therapy

Seventy older adults with a fall-related hip fracture were recruited from residential and rehabilitation center Zorggroep Solis in Deventer, The Netherlands between January 2012 and December 2014. An interim conditional power analysis conducted on the POMA indicated that 294 participants would be needed to demonstrate a significant intervention effect, which was not feasible within the study’s timeframe. The inclusion was therefore stopped before the planned number of 126 participants were included. Note that over 400 participants should have been included in the trial taking into account the dropout rate of 27% observed at T2. Of the 70 participants, 57 completed at least four weeks of training and were included in the analyses (Fig. 2 shows group distributions and reasons for dropout). The characteristics of the participants who dropped out were not significantly different from those who completed at least four weeks of training (all *p*>0.157), except that the participants who dropped out tended to be older and used more opioids (dropouts: 86.2±5.6 years, 50% used opioids; non-dropouts: 82.7±6.7 years, 19% used opioids, both *p*<0.080).

Table 2 shows the participant characteristics, which were not significantly different among the three training groups except for gender and the presence of urogenital disorders. No significant baseline differences in outcome measures were observed among groups (Tables 3 and 5). Out of the 30 specified training sessions, AT and CT groups performed respectively 11 (8–15) [median (minimum-maximum)] and 12 (7–14) training sessions on the treadmill and 13 (7–18) and 12 (7–15) sessions of usual physical therapy. The UPT group performed 27 (16–31) sessions of usual physical therapy. No serious adverse events related to the training sessions were reported.

**Table 2:** Participant characteristics

	AT (*n*=24)	CT (*n*=23)	UPT (*n*=23)	*P* value
Demographics				
Age, years	82.9 ± 6.5	83.9 ± 5.5	83.3 ± 8.0	0.877^a^
Height, m	168.6 ± 9.6, *n*=21	166.8 ± 10.8, *n*=22	166.2 ± 8.3	0.691^a^
Body mass, kg	72.3 ± 14.7, *n*=21	71.4 ± 12.7	68.7 ± 16.7	0.695^a^
Gender, n male (%)	8 (33.3)	9 (39.1)	2 (8.7)	**0.047** ^**b**^
Medical information				
Comorbidities	*n*=23			
Cardiovascular/respiratory diseases, n (%)	21 (91.3)	19 (82.6)	19 (82.6)	0.755^b^
Gastrointestinal disorders, n (%)	2 (8.7)	4 (17.4)	5 (21.7)	0.598^b^
Urogenital disorders, n (%)	6 (26.1)	3 (13.0)	0 (0.0)	**0.034** ^**b**^
Musculoskeletal disorders, n (%)	9 (39.1)	5 (21.7)	5 (21.7)	0.353^b^
Neurological disorders, n (%)	5 (21.7)	6 (26.1)	4 (17.4)	0.933^b^
Psychiatric disorders, n (%)	1 (4.3)	2 (8.7)	3 (13.0)	0.865^b^
Endocrine disorders, n (%)	6 (26.1)	8 (34.8)	11 (47.8)	0.346^b^
Other, n (%)	0 (0.0)	1 (4.3)	0 (0.0)	1.000^b^
Medication use	*n*=23			
Vitamine D, n (%)	6 (26.1)	5 (21.7)	9 (39.1)	0.503^b^
Antidepressants, n (%)	2 (8.7)	3 (13.0)	4 (17.4)	0.902^b^
Benzodiazepines, n (%)	3 (13.0)	3 (13.0)	3 (13.0)	1.000^b^
Antihypertensives, n (%)	17 (73.9)	15 (65.2)	16 (69.6)	0.945^b^
Analgesics: None/Non-opioids/Opioids, n (%)	1 (4.3)/16 (69.6)/6 (26.1)	1 (4.3)/15 (65.2)/7 (30.4)	0 (0.0)/19 (82.6)/4 (17.4)	0.754^c^
Type of fracture				0.814^b^
Cervical, n (%)	11 (45.8)	11 (47.8)	8 (34.8)	
Trochanteric, n (%)	12 (50.0)	12 (52.2)	14 (60.9)	
Subtrochanteric, n (%)	1 (4.2)	0 (0.0)	1 (4.3)	
Time since fracture, days^†^	13 (7–65)	13 (6–63)	14 (7–79)	0.924^c^
Surgery				0.610^b^
None, n (%)	0 (0.0)	0 (0.0)	2 (8.7)	
Total hip replacement, n (%)	1 (4.2)	0 (0.0)	1 (4.3)	
Hemiarthroplasty, n (%)	8 (33.3)	10 (43.5)	6 (26.1)	
Intramedullary nail, n (%)	11 (45.8)	10 (43.5)	9 (39.1)	
Dynamic hip screw, n (%)	4 (16.7)	2 (8.7)	5 (21.7)	
Other, n (%)	0 (0.0)	1 (4.3)	0 (0.0)	
Pre-fracture functioning				
Functional ambulation category, FAC [0–5]^†^	5.0 (4.0–5.0)	5.0 (4.0–5.0)	5.0 (2.0–5.0)	1.000^c^
Living situation, n living independent (%)	21 (87.5)	16 (70.0)	18 (78.3)	0.312^b^
Ambulatory assistance				
Indoors, n (%)	5 (20.8)	5 (21.7)	6 (26.1)	0.939^b^
Outdoors, n (%)	14 (58.3)	12 (52.2)	12 (52.2)	0.911^b^
Tolerated walking distance, n >1000 m (%)	8 (33.3)	9 (39.1)	14 (60.9)	0.138 ^b^
Baseline functioning				
Functional Ambulation Category, FAC [0–5]^†^	2.0 (2.0–4.0), *n*=23	2.0 (2.0–4.0)	2.0 (2.0–4.0)	0.200^c^
Performance oriented mobility assessment, POMA [0–28]	15.6 ± 2.8	15.5 ± 3.6	15.6 ± 4.1	0.994^a^
Mini-Mental State Examination, MMSE [0–30]^†^	25.0 (19.0–30.0), *n*=23	26.0 (22.0–30.0)	27.0 (22.0–29.0)	0.342^c^

Abbreviations: *AT*: adaptability treadmill training group, *CT*: conventional treadmill training group, *UPT*: usual physical therapy group

^†^Reported as median (minimum-maximum)

*P* values were obtained using

^a^One-Way ANOVA

^b^Fisher’s exact test

^c^Kruskal-Wallis test

Significant differences among groups are presented in bold (*p* < 0.05)

**Table 3:** General walking ability

Outcome (n: AT, CT, UPT)	INTERVENTION			Difference among groups		Mixed RM ANOVA T0, T1, T2		
	AT	CT	UPT	*P* value	Effect size (η_p_^2^)	Time effect	Group effect	Time × Group
POMA (0–28)								
T0 (19, 17, 21)	15.5 ± 2.9	15.6 ± 3.2	15.6 ± 4.4	0.997^a^	0.000	F (2)=83.423 *p*<0.001 η_p_^2^=0.635	F (2)=0.257 *p*=0.775 η_p_^2^=0.011	F (3.3)=0.484 *p*=0.712 η_p_^2^=0.020
T1 (19, 17, 21)	19.4 ± 2.1	19.6 ± 2.0	18.6 ± 3.5	0.188^b^	0.061			
T2 (17, 17, 17)	20.2 ± 2.4	20.6 ± 3.8	19.5 ± 3.7	0.444^b^	0.034			
T3 (8, 12, 14)	22.4 ± 3.8	23.6 ± 3.5	23.1 ± 3.6	0.367^b^	0.065			
EMS (0–20)								
T0 (19, 17, 21)	11.2 ± 3.7	11.1 ± 3.3	10.7 ± 3.7	0.904^a^	0.004	F (2)=98.133 *p*<0.001 η_p_^2^=0.672	F (2)=0.099 *p*=0.906 η_p_^2^=0.004	F (2.9)=1.072 *p*=0.374 η_p_^2^=0.043
T1 (19, 17, 21)	15.3 ± 2.3	16.5 ± 1.5	15.2 ± 3.2	0.191^b^	0.061			
T2 (17, 17, 17)	16.2 ± 2.6	16.3 ± 2.4	16.3 ± 3.5	0.912^b^	0.004			
T3 (8, 12, 14)	17.1 ± 2.9	18.1 ± 1.8	17.6 ± 2.3	0.334^b^	0.070			
TUG (s)								
T1 (19,17,21)	26.0 (13.5–60.7)	23.7 (13.6–48.9)	23.8 (13.3–79.9)	0.862^c^	-	Z=−4.134^d^ *p*<0.001 *r* = 0.400	-	*X* ^2^ (2)=3.835^d^ *p*=0.147
T2 (17,17,17)	19.9 (11.9–49.3)	23.6 (10.4–88.9)	22.2 (10.4–42.2)	0.871^c^	-			
FAC (0–5)								
T0 (19, 17, 21)	2.0 (2.0–4.0)	2.0 (2.0–4.0)	2.0 (2.0–4.0)	0.110^c^	-	*X* ^2^ (2)=81.0 *p*<0.001	-	-
ΔT1 (19, 17, 21)	1.0 (0.0–2.0)	2.0 (1.0–2.0)	1.0 (0.0–2.0)	**0.003** ^**c**^	-			
ΔT2 (17, 17, 17)	1.0 (0.0–2.0)	2.0 (0.0–3.0)	1.5 (0.0–3.0)	**0.039** ^**c**^	-			
ΔT3 (8, 12, 14)	1.5 (0.0–3.0)	2.0 (1.0–3.0)	2.0 (1.0–3.0)	0.417^c^	-			
Walking speed (m/s)								
T1 (19,17,21)	0.65 ± 0.22	0.74 ± 0.24	0.62 ± 0.20	0.219^a^	0.055	F (1)=7.421 *p*=0.009 η_p_^2^=0.134	F (2)=0.369 *p*=0.693 η_p_^2^=0.015	F (2)=3.053 *p*=0.057 η_p_^2^=0.113
T2 (17,17,17)	0.73 ± 0.20	0.72 ± 0.26	0.72 ± 0.25	0.984^a^	0.001			
NEADL (0–66)								
T0 (18, 17, 21)	44.2 ± 9.7	42.5 ± 13.2	49.6 ± 10.9	0.133^a^	0.073	F (1)=28.180 *p*<0.001 η_p_^2^=0.370	F (2)=0.700 *p*=0.501 η_p_^2^=0.028	F (2)=2.828 *p*=0.069 η_p_^2^=0.105
T2 (17, 17, 17)	32.2 ± 16.4	36.9 ± 15.2	33.4 ± 18.6	0.267^b^	0.055			
T3 (8, 12, 14)	42.9 ± 11.7	42.0 ± 12.1	43.4 ± 11.5	0.748^b^	0.019			

Measures related to mobility, walking and daily functioning in the adaptability treadmill (AT) group, conventional treadmill (CT) group and usual physical therapy (UPT) group at baseline (T0), directly after (T1), four weeks after (T2) and 12 months (T3) after the intervention

*P* values for group differences were obtained using

^a^One-Way ANOVA

^b^ANCOVA with baseline performance as covariate

^c^Kruskal-Wallis test

^d^The effect of time was analyzed using Wilcoxon signed rank test and the Time × Group effect using Kruskal-Wallis test over the change score from T1 to T2.

Δ indicates change relative to baseline, which was evaluated over time using a Friedman test

Significant differences among groups are presented in bold (*p* < 0.05)

### Walking ability

#### General walking ability

All measures of general walking ability improved significantly over time (all *p*<0.032, Table 3), with the greatest improvements occurring during the intervention period. The performance of the POMA, EMS, TUG, 10MWT and NEADL was not significantly different among groups at any of the follow-up measurements (all *p*>0.133), and no significant Time × Group effects were observed in the mixed repeated measures ANOVAs (all *p*>0.057). Significant differences among groups were only revealed for FAC score at T1 (*p*=0.003) and T2 (*p*=0.039). Post hoc tests revealed that the improvement in FAC from T0 to T1 was significantly greater in the CT group than in AT and UPT groups (both *U*≤93.5, *p*<0.004, *r*>0.473). The improvement in FAC from T0 to T2 showed trends towards greater improvements in the CT group than in AT and UPT groups (*U*=81.5, *p*=0.016, *r*=0.418 and *U*=92.5, *p*=0.05, *r*=0.341, respectively).

#### Walking adaptability

The results of obstacle avoidance performance and dual-task walking are shown in Table 4. A significant difference among groups was only found for the dual-task effect on walking speed at T1 (*p*=0.046). Post hoc tests revealed trends towards smaller dual-task declines on walking speed in AT than in CT and UPT groups at T1 (*t* (33)=2.462, *p*=0.017, *r*=0.394 and *t* (37)=1.847, *p*=0.070, *r*=0.291, respectively).

**Table 4:** Walking adaptability

Outcome (n: AT, CT, UPT)	INTERVENTION			Difference among groups		Mixed RM ANOVA T0, T1, T2		
	AT	CT	UPT	P value	Effect size (η_p_^2^)	Time effect	Group effect	Time × Group
Obstacle effect (%)								
T1 (19,17,21)	−18.00 ± 10.96	−21.06 ± 11.17	−24.34 ± 14.45	0.281^a^	0.046	F (1)=0.907 *p*=0.346 η_p_^2^=0.019	F (2)=0.449 *p*=0.641 η_p_^2^=0.018	F (2)=0.750 *p*=0.478 η_p_^2^=0.030
T2 (17,17,17)	−21.48 ± 9.75	−24.38 ± 12.11	−21.63 ± 12.87	0.717^a^	0.014			
Obstacle success rate (%)								
T1 (19,17,21)	100.0 (16.7–100.0)	100.0 (0.0–100.0)	83.3 (0.0–100.0)	0.132^b^	-	Z=−1.744^b^ *p*=0.082 *r*=0.168	-	*X* ^2^ (2)=1.609^b^ *p*=0.447
T2 (17,17,17)	100.0 (33.3–100.0)	100.0 (16.7–100.0)	100.0 (33.3–100.0)	0.754^b^	-			
DTE- walking speed (%)								
T1 (18,17,21)	−21.48 ± 18.13	−35.91 ± 18.56	−31.77 ± 15.52	**0.046** ^**a**^	0.110	F (1)=3.115 *p*=0.084 η_p_^2^=0.062	F (2)=3.087 *p*=0.055 η_p_^2^=0.116	F (2)=2.779 *p*=0.072 η_p_^2^=0.106
T2 (16,17,17)	−22.85 ± 17.03	−32.20 ± 17.48	−19.83 ± 15.65	0.091^a^	0.097			
DTE- subtractions (%)								
T1 (17,17,21)	4.8 (−100.0–208.0)	0.0 (−62.8–55.6)	−4.8 (−100.0–58.8)	0.776^b^	-	Z= −0.413^b^ *p*=0.686 *r*=0.040	-	*X* ^2^ (2)=3.771^b^ p=0.152
T2 (16,17,17)	−7.3 (−100.0–120.2)	13.3 (−26.2–77.3)	9.4 (−100.0–677.9)	0.244^b^	-			

Measures related to obstacle avoidance performance and dual-task effects (DTE) in the adaptability treadmill (AT) group, conventional treadmill (CT) group and usual physical therapy (UPT) group directly after (T1) and four weeks after (T2) intervention

*P* values for group differences were obtained using

^a^One-Way ANOVA

^b^Kruskal-Wallis test with the effect of time analyzed using Wilcoxon signed rank test and the Time × Group effect using Kruskal-Wallis test over the change score from T1 to T2

Significant differences among groups are presented in bold (*p* < 0.05)

### Fear of falling and general health

As shown in Table 5, no significant changes among groups were found for FES-I, VAS, HOOS-Q, TMTa and TMTb (all *p*>0.117). The mixed repeated measures ANOVAs only revealed significant main effects of time for all measures (all *p*<0.032).

**Table 5:** Fear of falling and general health

Outcome (n: AT, CT, UPT)	INTERVENTION			Difference among groups		Mixed RM ANOVA T0, T1, T2		
	AT	CT	UPT	P value	Effect size (η_p_^2^)	Time effect	Group effect	Time × Group
FES-I (20–80)								
T0 (19,17,21)	44.68 ± 12.32	44.71 ± 13.00	46.52 ± 14.78	0.885^a^	0.005	F (1.6)=4.001 *p*=0.031 η_p_^2^=0.077	F (2)=0.562 *p*=0.574 η_p_^2^=0.023	F (3.2)=1.424 *p*=0.241 η_p_^2^=0.056
T1 (19,17,21)	44.16 ± 15.86	36.24 ± 13.70	39.95 ± 15.06	0.212^b^	0.057			
T2 (17,17,17)	41.94 ± 14.29	37.94 ± 14.37	39.24 ± 17.20	0.681^b^	0.016			
T3 (8,12,14)	31.63 ± 15.73	28.58 ± 5.92	27.50 ± 6.53	0.500^b^	0.045			
VAS (0–100)								
T0 (19,17,21)	61.89 ± 18.23	67.85 ± 18.88	60.57 ± 20.94	0.491^a^	0.026	F (1.6)=21.945 *p*<0.001 η_p_^2^=0.314	F (2)=0.968 *p*=0.387 η_p_^2^=0.039	F (3.2)=0.272 *p*=0.859 η_p_^2^=0.011
T1 (18,17,21)	80.83 ± 16.02	80.62 ± 15.88	76.29 ± 16.39	0.661^b^	0.016			
T2 (17,17,17)	80.82 ± 18.40	80.94 ± 14.81	76.26 ± 16.46	0.805^b^	0.009			
T3 (8,12,13)	78.13 ± 17.31	82.88 ± 11.39	77.65 ± 12.24	0.675^b^	0.027			
HOOS-Q (0–100)								
T2 (17,17,17)	54.78 ± 30.10	57.35 ± 23.41	50.37 ± 20.78	0.715^a^	0.014	n: 8, 12, 12 F (1)=19.876 *p*<0.001 η_p_^2^=0.407	n: 8, 12, 12 F (2)=0.642 *p*=0.534 η_p_^2^=0.042	n: 8, 12, 12 F (2)=1.840 *p*=0.177 η_p_^2^=0.113
T3 (8,12,14)	76.56 ± 23.80	70.31 ± 23.10	78.57 ± 19.26	0.619^a^	0.030			
TMTa (s)								
T0 (19, 17, 21)	98.0 (41.0–262.0)	100.0 (47.2–164.0)	86.0 (27.0–300.0)	0.870^c^	-	F (1.6)=6.38 *p*=0.005 η_p_^2^=0.120	F (2)=0.081 *p*=0.922 η_p_^2^=0.003	F (3.1)=2.296 *p*=0.083 η_p_^2^=0.089
T1 (19, 17, 21)	88.8 ± 42.1	83.4 ± 38.2	86.3 ± 58.6	0.614^b^	0.018			
T2 (16, 17, 17)	78.8 ± 39.2	97.8 ± 71.1	74.2 ± 49.3	0.117^b^	0.089			
T3 (8, 12, 14)	56.8 ± 14.9	80.9 ± 40.9	84.1 ± 40.5	0.615^b^	0.032			
TMTb (s)								
T0 (18, 17, 21)	205.0 (100.0–300.0)	252.0 (82.6–300.0)	232.0 (80.0–300.0)	0.747^c^	-	F (2)=3.564 *p*=0.032 η_p_^2^=0.072	F (2)=0.251 *p*=0.779 η_p_^2^=0.011	F (4)=0.266 *p*=0.899 η_p_^2^=0.011
T1 (18, 17, 21)	217.4 ± 87.9	211.9 ± 78.4	200.6 ± 88.0	0.479^b^	0.028			
T2 (15, 17, 17)	198.1 ± 90.0	207.0 ± 82.8	191.5 ± 96.2	0.731^b^	0.014			
T3 (7, 12, 14)	200.6 ± 78.2	202.8 ± 90.7	207.3 ± 94.8	0.246^b^	0.092			

Measures related to fear of falling and general health in the adaptability treadmill (AT) group, conventional treadmill (CT) group and usual physical therapy (UPT) group at baseline (T0), directly after (T1), four weeks after (T2) and 12 months (T3) after the intervention

*P* values for group differences were obtained using

^a^One-Way ANOVA

^b^ANCOVA with baseline performance as covariate

^c^Kruskal-Wallis test

### Falls

Forty-six participants monitored their falls for at least 6 months after T1. Eighteen participants (39%) reported 42 falls during an average time of 11.5 months. Table 6 shows the distribution of falls over groups along with the calculated incidence rates and number (proportion) of fallers. Incidence rate ratios and relative risks were calculated relative to UPT, and were respectively 0.63 (95% CI: 0.22–1.77, *p*=0.377) and 0.51 (95% CI: 0.20–1.29, *p*=0.159) for AT training and 0.59 (95% CI: 0.22–1.64, *p*=0.314) and 0.56 (95% CI: 0.24–1.29, *p*=0.285) for CT training.

**Table 6:** Falls in the three intervention groups during the 12-month follow-up period

	AT (n=14)	CT (n=16)	UPT (n=16)
Number of falls	11	11	20
Observation time, person-years	13.51	15.14	15.49
Fall incidence rate, falls/person-years	0.81	0.77	1.29
Number (%) of fallers	4 (28.6)	5 (31.3)	9 (56.3)
Incidence rate ratio (95% CI), relative to UPT	0.63 (0.22–1.76, *p*=0.377)	0.59 (0.22–1.64, *p*=0.314)	
Relative risk (95% CI), relative to UPT	0.51 (0.20–1.29, *p*=0.159)	0.56 (0.24–1.29, *p*=0.285)	

Abbreviations: *AT*: adaptability treadmill, *CT*: conventional treadmill, *UPT*: usual physical therapy, *CI*: confidence interval

## Discussion

The present study showed that measures of walking ability, fear of falling and general health improved over time in all groups, which is consistent with previous studies showing improved walking ability after exercise programs in older adults with hip fracture [38, 40, 41]. Overall, similar improvements were observed for adaptability treadmill training, conventional treadmill training and usual physical therapy. Significant group differences were only found for the Functional Ambulation Category and the dual-task effect on walking speed.

Functional Ambulation Category scores improved significantly more from baseline to post-intervention assessments after conventional treadmill training than after usual physical therapy and adaptability treadmill training. The non-significantly lower baseline Functional Ambulation Category scores in the group receiving conventional treadmill training might have contributed to this intervention effect. More interestingly, a task-specific intervention effect on the dual-task effect on walking speed was found in favor of adaptability treadmill training. Previous intervention studies on adaptive walking or stepping, combining aspects of motor and cognitive practice, also showed reductions in dual-task effects for both simple and adaptive walking [13, 42, 43], while dual-task effects remained unchanged after resistance training and Tai Chi training, for example [44–46]. Improvements in the dual-task effects on walking thus appear highly specific to the performed intervention, which should preferably combine aspects of motor and cognitive practice such as with adaptability treadmill training. This is especially important for the participants included in the present study, who showed reduced physical functioning as well as reduced cognitive and executive functioning (see Table 2). Interestingly, cognitive dual tasks constitute an important aspect of the construct of walking adaptability [47].

The present study further revealed clinically relevant reductions in fall rate and the proportion of fallers after adaptability and conventional treadmill training relative to usual physical therapy (37% - 49% reduction). Although these reductions in fall rate and the proportion of fallers did not reach significance, presumably due to a not sufficiently high sample size, they match fairly well with previous research showing a 46% reduction in fall rate after an overground walking adaptability program in older adults [7] as well as with a recent review showing that stepping interventions in response to environmental challenges reduced fall rate by 52% and the proportion of fallers by 49% [9]. These encouraging results notwithstanding, we found no compelling evidence that conventional or adaptability treadmill training is more effective in improving walking ability and in reducing fear of falling and fall incidence than usual physical therapy after hip fracture in older adults. Several possible reasons for this null effect are discussed below.

First, although adaptability treadmill training aimed to improve walking adaptability, our outcome measures may not have sufficiently covered this construct. In contrast to the present study, Weerdesteyn et al. [7] and Yamada et al. [8] showed better obstacle avoidance performance after overground interventions incorporating walking adaptability exercises in older adults. Obstacle avoidance in these studies was evaluated under very demanding conditions (e.g., obstacles suddenly appeared under high time pressure demands), which may have better covered the construct of walking adaptability than the rather simple obstacle avoidance task used in the present study. This suggestion is supported empirically by Yamada et al. [8], who reported significant differences between two obstacle course interventions on performance time and obstacle failure rate in a complex obstacle course but not in a simple obstacle course. Moreover, previous research showed differences between older and young adults in success rates of time-constrained obstacle avoidance but not for time-unconstrained obstacle avoidance as used in the present study [5]. Walking adaptability is a complex and multifaceted construct that involves more than simple obstacle avoidance [47]. A gold standard clinical assessment that covers all aspects of walking adaptability seems necessary and should, if feasible, be included as outcome measure in future research on walking adaptability [47, 48].

Second, the provided adaptability treadmill training did not address all aspects of walking adaptability, which may have hampered its efficacy. Although obstacle negotiation, time constraints and speeding up and slowing down were practiced, other important aspects of walking adaptability were not directly practiced; walking while performing motor dual tasks, cognitive dual tasks, postural transitions such as turning and bending, walking around objects, walking on uneven surfaces such as stairs, ramps and grass, walking under different ambient conditions and walking with physical load [47]. Interventions that incorporate all aspects of walking adaptability may be more effective in improving this skill and reducing fall incidence and fear of falling.

Third, our three interventions differed only by five hours of scheduled practice. This moderate contrast among the three intervention programs might have contributed to the absence of convincing intervention effects. Moreover, all our intervention groups received active training aimed at improving walking ability, unlike for example the interventions in the study by Hauer et al. [37, 38], on which we based our sample size calculation. It appears that the contrast among our three intervention groups was too small to detect intervention effects, given this heterogeneous and multi-morbid population.

The dropout rate in the present study was high (51.4% over the complete follow-up period), with participants dropping out for reasons as diverse as early discharge home, illness, death and experienced stress with participation (Fig. 2). Although the high dropout rate was not related to the interventions, it reduced the study’s power, which was already limited by the relatively small sample size. However, the sizes of group effects were only small to medium and confirmed our findings that the three interventions effects differed little overall. The bias caused by dropouts may have reduced the generalizability of the results, because dropouts may have responded differently to the interventions. The generalization of results to a wide range of older adults is further reduced by including a specific group of older adults with a recent fall-related hip fracture admitted to a residential and rehabilitation center after hospital discharge and by analyzing the study’s data per protocol, which does not reflect the clinical practice where noncompliance and protocol deviations occur.

**Fig. 3:**
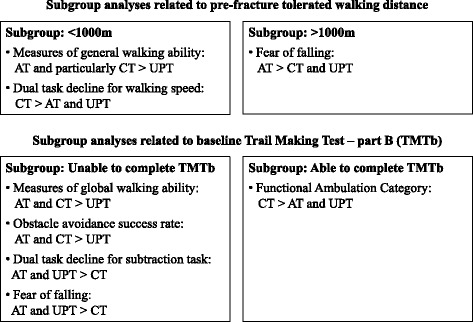
Overview of the significant main intervention effects (*p*<0.05) observed in the four subgroups. Abbreviations: AT: adaptability treadmill, CT: conventional treadmill, UPT: usual physical therapy, TMTb: trail making test part B

Together with the considerable heterogeneity and multi-morbidity in our sample of frail older adults (83.3±6.7 years), the low power may have hampered the detection of intervention effects for the entire group. Subgroup analyses might therefore be useful because this will reduce within-group variations. In addition, intervention effects might be particularly present for certain subgroups. For example, since the two treadmill-based interventions evoked more than twice as many steps than usual physical therapy [15], the two treadmill-based interventions might be particularly useful for improving general walking ability in participants who did not perform many steps before their hip fracture. Further, adaptability treadmill training might be particularly useful for improving walking adaptability in participants with reduced cognitive functioning, since walking adaptability strongly relies on cortical control [47]. We therefore performed additional post hoc subgroup analyses to explore possible differential intervention effects relating to pre-fracture tolerated walking distance (>1000 m vs. ≤1000 m) and executive function (able vs. unable to complete TMTb in five minutes at baseline). Subgroup analyses revealed several significant main intervention effects, a schematic presentation of which is shown in Fig. 3 (See Additional files 1 and 2 for detailed results). Trends and significant intervention effects were particularly evident in subgroups with low tolerated walking distance and low executive function, and were generally in favor of conventional and adaptability treadmill training.

These results indicate that conventional and adaptability treadmill training might be more effective compared to usual physical therapy in certain subgroups. For clinical practice, this means that adaptability and conventional treadmill training have no added value over usual physical therapy for the entire group of older adults with a fall-related hip fracture, but may be useful for certain subgroups. Although the subgroup analyses in the present study were well motivated and plausible, subgroup analyses pose multiplicity concerns and increase the risk of false-positives (i.e., type-I errors), which requires careful interpretation of their results. The limitations notwithstanding, we believe that our study provides important information to be implemented in a larger sample size study. Specifically, for future research we recommend well-powered studies with pre-specified subgroups to univocally identify the characteristics of individuals who are likely to respond well to conventional and adaptability treadmill training. Such studies are advised to provide sufficient contrast among the intervention groups and to include a gold standard clinical assessment that covers a broader range of walking adaptability aspects.

## Conclusions

Overall, adaptability treadmill training, conventional treadmill training and usual physical therapy led to similar effects on walking ability, fear of falling and fall incidence in older adults rehabilitating from a fall-related hip fracture. With the exception of a task-specific reduction in dual-task decline on walking speed after adaptability treadmill training, the use of a treadmill with or without walking adaptability exercises did not lead to notably better outcomes in our heterogeneous, frail and multi-morbid sample. Post hoc subgroup analyses, however, revealed several intervention effects that were generally in favor of conventional and adaptability treadmill training. Future research with pre-specified subgroup analyses is needed for identifying the characteristics of individuals who respond best to adaptability and conventional treadmill training.

## Additional files

Additional file 1: Results of the subgroup analyses related to tolerated pre-fracture walking distance. (PDF 189 kb)
Additional file 2: Results of the subgroup analyses related to the ability to perform Trail Making Test part B (TMTb) at baseline. (PDF 195 kb)
Additional file 3: Individual participant data regarding walking ability, fear of falling, general health and falls. (XLSX 31 kb)

## Abbreviations

10MWT: 10 meter walk test
ANCOVA: Analysis of covariance
ANOVA: Analysis of variance
AT: Adaptability treadmill
CI: Confidence interval
CT: Conventional treadmill
DTE: Dual-task effects
EMS: Elderly mobility scale
FAC: Functional ambulation category
FES-I: Falls efficacy scale international
HOOS-Q: Hip disability and Osteoarthritis Score - subscale Quality of life
LSD: Least significant difference
MMSE: Mini-mental state examination
NEADL: Nottingham extended activities of daily living
POMA: Performance oriented mobility scale
T0: Pre-intervention assessment
T1: Post-intervention assessment
T2: 4-week follow-up assessment
T3: 12-month follow-up assessment
TMTa: Trail making test part A
TMTb: Trail making test part B
TUG: Timed up-and-go
UPT: Usual physical therapy
VAS: Visual analogue scale
