# Supplementary material for: The efficacy of treadmill training with and without projected visual context for improving walking ability and reducing fall incidence and fear of falling in older adults with fall-related hip fracture: a randomized controlled trial
Source: BMC Geriatr. 2016 Dec 28;16:215. doi: 10.1186/s12877-016-0388-x (PMC5198499; doi:10.1186/s12877-016-0388-x)
Supplement: Additional file 1: — Results of the subgroup analyses related to tolerated pre-fracture walking distance. (PDF 189 kb) [file 12877_2016_388_MOESM1_ESM.pdf]

.

SUBGROUP: > 1000m					
**Outcome (n: AT,CT,UPT)**	**INTERVENTION**			**Difference among groups**	
	**AT**	**CT**	**UPT**	***P* value**	**Effect size (η_p^2)**
**POMA (0-28)**					
T0 (5, 7, 13)	16.4 ± 3.1	15.14 ± 2.4	16.6 ± 3.5	0.608[a]	0.044
T1 (5, 7, 13)	18.8 ± 1.5	19.0 ± 2.4	19.4 ± 3.0	0.780[b]	0.023
T2 (4, 7, 11)	18.5± 3.9	19.6 ± 4.5	20.4 ± 3.3	0.686[b]	0.041
T3 (3, 5, 8)	20.7 ± 5.8	22.8 ± 4.7	25.1 ± 2.1	0.119[b]	0.298
**EMS (0-20)**					
T0 (5, 7, 13)	12.6 ± 3.1	10.6 ± 2.6	11.5 ± 3.9	0.613[a]	0.044
T1 (5, 7, 13)	14.2 ± 3.3	16.6 ± 1.6	16.4 ± 2.5	0.102[b]	0.196
T2 (4, 7, 11)	15.8 ± 3.2	15.6 ± 3.2	17.6 ± 2.5	0.315[b]	0.121
T3 (3, 5, 8)	17.3 ± 4.6	17.4 ± 2.4	17.9 ± 2.0	0.859[b]	0.018
**TUG (s)**					
T1 (5, 7, 13)	32.4 (17.8-37.5)	21.6 (14.5-48.9)	21.9 (14.5-36.5)	0.504[c]	-
T2 (4, 7, 11)	23.2 (15.4-27.3)	23.8 (15.1-37.9)	22.2 (10.4-34.1)	0.580[c]	-
**FAC (0-5)**					
T0 (5, 7, 13)	2.0 (2.0-3.0)	2.0 (2.0-4.0)	2.0 (2.0-4.0)	0.570[c]	-
ΔT1 (5, 7, 13)	1.0 (1.0-2.0)	2.0 (1.0-2.0)	1.0 (-1.0-2.0)	0.304[c]	-
ΔT2 (4, 7, 11)	1.0 (1.0-2.0)	2.0 (0.0-2.0)	1.0 (0.0-2.0)	0.829[c]	-
ΔT3 (3, 5, 8)	2.0 (1.0-3.0)	2.0 (1.0-3.0)	2.0 (1.0-3.0)	0.943[c]	-
**Walking speed (m/s)**					
T1 (5, 7, 13)	0.58 ± 0.25	0.69 ± 0.25	0.67 ± 0.17	0.661[a]	0.037
T2 (4, 7, 11)	0.69 ± 0.14	0.67 ± 0.22	0.75 ± 0.22	0.724[a]	0.033
**NEADL (0-66)**					
T0 (5, 7, 13)	52.4 ± 3.4	50.4 ± 13.5	54.1 ± 6.9	0.681[a]	0.034
T2 (4 7, 11)	31.9 ± 20.9	29.6 ± 17.0	34.8 ± 16.8	0.962[b]	0.004
T3 (3, 5, 8)	41.0 ± 19.2	40.6 ± 16.6	46.1 ± 9.8	0.863[b]	0.024

Measures related to mobility, walking and daily functioning in the adaptability treadmill (AT) group, conventional treadmill (CT) group and usual physical therapy (UPT) group at baseline (T0), directly after (T1), four weeks after (T2) and 12 months (T3) after the intervention. *P* values for group differences were obtained using [a] One-Way ANOVA, [b] ANCOVA with baseline performance as covariate and [c]Kruskal-Wallis test.

SUBGROUP: < 1000m					
**Outcome (n: AT,CT,UPT)**	**INTERVENTION**			**Difference among groups**	
	**AT**	**CT**	**UPT**	***P* value**	**Effect size (η_p^2)**
**POMA (0-28)**					
T0 (14, 10, 8)	15.2 ± 2.9	15.9 ± 3.8	14.0 ± 5.3	0.589[a]	0.036
T1 (14, 10, 8)	19.6 ± 2.3 *	20.1 ± 1.7 *	17.3 ± 4.1	0.086[b]	0.160
T2 (13, 10, 6)	20.8 ± 1.5	21.4 ± 3.2	17.8 ± 4.2	0.116[b]	0.158
T3 (5, 7, 6)	23.4 ± 2.3	24.1 ± 2.5 *	20.3 ± 3.6	0.059[b]	0.333
**EMS (0-20)**					
T0 (14, 10, 8)	10.7 ± 3.9	11.4 ± 3.8	9.5 ± 3.2	0.558[a]	0.039
T1 (14, 10, 8)	15.6 ± 1.9 *	16.4 ± 1.6 *	13.3 ± 3.4	**0.033[b]**	0.216
T2 (13, 10, 6)	16.3 ± 2.5	16.8 ± 1.8	14.0 ± 4.1	0.244[b]	0.107
T3 (5, 7, 6)	17.0 ± 1.9	18.6 ± 1.3	17.2 ± 2.1	0.207[b]	0.202
**TUG (s)**					
T1 (14, 10, 8)	25.8 (13.5-60.7)	24.6 (13.6-37.7)	27.2 (13.3-79.9)	0.571[c]	-
T2 (13, 10, 6)	19.3 (11.9-49.3)	20.3 (10.4-88.9)	27.9 (12.9-42.2)	0.839[c]	-
**FAC (0-5)**					
T0 (14, 10, 8)	2.5 (2.0-4.0) †	2.0 (2.0-2.0)	2.0 (2.0-3.0)	**0.014[c]**	-
ΔT1 (14, 10, 8)	1.0 (0.0-2.0) ††	2.0 (2.0-2.0)**	1.0 (0.0-2.0)	**0.010[c]**	-
ΔT2 (13, 10, 6)	1.0 (0.0-2.0) ††	2.0 (2.0-3.0)	1.5 (0.0-3.0)	**0.021[c]**	-
ΔT3 (5, 7, 6)	1.0 (0.0-3.0)	2.0 (2.0-3.0)	2.0 (2.0-3.0)	0.148[c]	-
**Walking speed (m/s)**					
T1 (14, 10, 8)	0.67 ± 0.22	0.78 ± 0.25	0.56 ± 0.25	0.149[a]	0.123
T2 (13, 10, 6)	0.75 ± 0.22	0.75 ± 0.28	0.67 ± 0.31	0.757[a]	0.021
**NEADL (0-66)**					
T0 (13, 10, 8)	41.0 ± 9.5	36.9 ± 10.1	42.3 ± 12.6	0.518[a]	0.046
T2 (13, 10, 6)	32.4 ± 15.8 †	42.1 ± 12.2**	30.8 ± 23.1	**0.006[b]**	0.336
T3 (5, 7, 6)	44.0 ± 7.1	43.0 ± 9.1	39.7 ± 13.5	0.355[b]	0.137

Measures related to mobility, walking and daily functioning in the adaptability treadmill (AT) group, conventional treadmill (CT) group and usual physical therapy (UPT) group at baseline (T0), directly after (T1), four weeks after (T2) and 12 months (T3) after the intervention. *P* values for group differences were obtained using [a] One-Way ANOVA, [b] ANCOVA with baseline performance as covariate and [c]Kruskal-Wallis test.

Significant group effects are presented in bold (p<0.05). Post-hoc tests:

** Significantly different from usual physical therapy (UPT) (p<0.01)

* Trend towards different from usual physical therapy (UPT) (p<0.05)

†† Significantly different from conventional treadmill training (CT) (p<0.01)

† Trend towards different from conventional treadmill training (CT) (p<0.05)

SUBGROUP: > 1000m					
**Outcome (n: AT, CT, UPT)**	**INTERVENTION**			**Difference among groups**	
	**AT**	**CT**	**UPT**	**P value**	**Effect size (η_p^2)**
**Obstacle effect (%)**					
T1 (5, 7, 13)	-20.4 ± 13.8	-17.1 ± 10.7	-27.7 ± 15.4	0.258[a]	0.116
T2 (4, 7, 11)	-27.0 ± 7.0	-23.5 ± 10.6	-21.2 ± 10.6	0.623[a]	0.049
**Obstacle success rate (%)**					
T1 (5, 7, 13)	100.0 (66.7-100.0)	83.3 (0.0-100.0)	83.3 (0.0-100.0)	0.403[b]	-
T2 (4, 7, 11)	100.0 (66.7-100.0)	100.0 (33.3-100.0)	86.4(33.3-100.0)	0.931[b]	-
**DTE- walking speed (%)**					
T1 (5, 7, 13)	-18.0 ± 26.0	-28.6 ± 21.1	-37.7 ± 10.1	0.109[a]	0.182
T2 (4, 7, 11)	-24.3 ± 28.6	-38.0 ± 18.9	-24.6 ± 17.2	0.353[a]	0.104
**DTE- subtractions (%)**					
T1 (5, 7, 13)	4.8 (-100.0-48.7)	0.0 (-30.1-26.8)	-0.2 (-29.7-48.0)	0.941[b]	-
T2 (4, 7, 11)	-14.1(-100.0-36.4)	0 (-12.9-59.7)	9.4 (-46.0-677.9)	0.641[b]	-

Measures related to obstacle avoidance performance and dual-task effects (DTE) in the adaptability treadmill (AT) group, conventional treadmill (CT) group and usual physical therapy (UPT) group directly after (T1) and four weeks after (T2) intervention. *P* values for group differences were obtained using [a] One-Way ANOVA and [b] Kruskal-Wallis test.

SUBGROUP: < 1000M					
**Outcome (n: AT, CT, UPT)**	**INTERVENTION**			**Difference among groups**	
	**AT**	**CT**	**UPT**	**P value**	**Effect size (η_p^2)**
**Obstacle effect (%)**					
T1 (14, 10, 8)	-17.1 ± 10.2	-23.9 ± 11.1	-18.9 ± 11.5	0.330[a]	0.074
T2 (13, 10, 6)	-19.8 ± 10.1	-25.0 ± 13.6	-22.4 ± 17.4	0.637[a]	0.034
**Obstacle success rate (%)**					
T1 (14, 10, 8)	100.0 (16.7-100.0)	100.0 (50.0-100.0)	75.0(0.0-100.0)	0.198[b]	-
T2 (13, 10, 6)	100.0 (33.3-100.0)	91.7 (16.7-100.0)	91.7 (66.7-100.0)	0.696[b]	-
**DTE- walking speed (%)**					
T1 (13, 10, 8)	-22.8 ± 15.3 †	-41.0 ± 15.7 *	-22.2 ± 18.6	**0.023[a]**	0.237
T2 (12, 10, 6)	-22.4 ± 13.1	-28.1 ± 16.1 *	-11.1 ± 7.4	0.067[a]	0.195
**DTE- subtractions (%)**					
T1 (12, 10, 8)	5.1 (-100.0-208.0)	2.0 (-62.8-55.6)	-22.1 (-100.0-58.8)	0.488[b]	-
T2 (12, 10, 6)	-3.9 (-100.0-120.2)	20.8 (-26.2-77.3)	-10.9 (-100.0-31.3)	0.256[b]	-

Measures related to obstacle avoidance performance and dual-task effects (DTE) in the adaptability treadmill (AT) group, conventional treadmill (CT) group and usual physical therapy (UPT) group directly after (T1) and four weeks after (T2) intervention. *P* values for group differences were obtained using [a] One-Way ANOVA and [b] Kruskal-Wallis test.
Significant group effects are presented in bold ($p<0.05$). Post-hoc tests:
* Trend towards different from usual physical therapy (UPT) ($p<0.05$)
† Trend towards different from conventional treadmill training (CT) ($p<0.05$)

SUBGROUP: > 1000m					
**Outcome (n: AT, CT, UPT)**	**INTERVENTION**			**Difference among groups**	
	**AT**	**CT**	**UPT**	**P value**	**Effect size (η_p^2)**
**FES-I (20-80)**					
T0 (5, 7, 13)	40.6 ± 7.1	49.6 ± 12.7	45.5 ± 16.5	0.565[a]	0.051
T1 (5, 7, 13)	50.4 ± 21.1 * ††	31.9 ± 12.0	34.5 ± 11.2	**0.015[b]**	0.331
T2 (4, 7, 11)	53.0 ± 15.3 * †	36.6 ± 11.7	34.7 ± 12.6	0.057[b]	0.273
T3 (3, 5, 8)	43.3 ± 21.5 *	28.8 ± 5.8	24.8 ± 3.5	**0.046[b]**	0.401
**VAS (0-100)**					
T0 (5, 7, 13)	71.5 ± 14.8	72.9 ± 18.3	62.7 ± 18.7	0.418[a]	0.076
T1 (5, 7, 13)	85.0 ± 15.0	75.6 ± 19.7	78.1 ± 17.8	0.667[b]	0.038
T2 (4, 7, 11)	87.3 ± 11.7	75.7 ± 13.7	82.1 ± 15.3	0.337[b]	0.114
T3 (3, 5, 7)	66.7 ± 15.3	83.7 ± 13.7	76.4 ± 12.5	0.129[b]	0.311
**HOOS-Q (0-100)**					
T2 (4, 7, 11)	40.6 ± 23.7	50.0 ± 24.5	55.7 ± 23.8	0.565[a]	0.058
T3 (3, 5, 8)	62.5 ± 33.1	78.8 ± 25.6	83.6 ± 18.0	0.432[a]	0.121
**TMTa (s)**					
T0 (5, 7, 13)	76.2 (48.8-148.0)	101.6 (50.8-127.0)	105.0 (49.0-177.0)	0.436[c]	-
T1 (5, 7, 13)	75.1 ± 32.0	96.5 ± 54.0	80.5 ± 36.8	0.358[b]	0.093
T2 (4, 7, 11)	59.4 ± 20.0	116.9 ± 85.7 *	64.3 ± 20.6	0.092[b]	0.233
T3 (3, 5, 8)	62.1 ± 4.7	81.5 ± 52.5	84.1 ± 40.5	0.958[b]	0.007
**TMTb (s)**					
T0 (5, 7, 13)	182.0 (100.0-300.0)	300.0 (110.8-300.0)	247.0 (80.0-300.0)	0.213[c]	-
T1 (5, 7, 13)	188.3 ± 94.9	236.7 ± 75.7	193.2 ± 81.1	0.653[b]	0.040
T2 (4, 7, 11)	177.0 ± 89.1	219.6 ± 82.5	189.4 ± 90.3	0.895[b]	0.012
T3 (3, 5, 8)	212.3 ± 92.1	193.8 ± 86.3	215.0 ± 92.9	0.348[b]	0.161

Measures related to fear of falling and general health in the adaptability treadmill (AT) group, conventional treadmill (CT) group and usual physical therapy (UPT) group at baseline (T0), directly after (T1), four weeks after (T2) and 12 months (T3) after the intervention. *P* values for group differences were obtained using [a] One-Way ANOVA, [b] ANCOVA with baseline performance as covariate and [c] Kruskal-Wallis test.
Significant group effects are presented in bold ($p<0.05$). Post-hoc tests:
* Trend towards different from usual physical therapy (UPT) ($p<0.05$)
†† Significantly different from conventional treadmill training (CT) ($p<0.01$)
† Trend towards different from conventional treadmill training (CT) ($p<0.05$)

SUBGROUP: < 1000m					
**Outcome (n: AT, CT, UPT)**	**INTERVENTION**			**Difference among groups**	
	**AT**	**CT**	**UPT**	**P value**	**Effect size (η_p^2)**
**FES-I (20-80)**					
T0 (14, 10, 8)	46.1 ± 13.6	41.3 ± 12.7	48.1 ± 12.4	0.514[a]	0.045
T1 (14, 10, 8)	41.9 ± 13.8	39.3 ± 14.5	48.8 ± 17.1	0.586[b]	0.037
T2 (13, 10, 6)	38.5 ± 12.7	38.9 ± 16.5	47.5 ± 22.4	0.497[b]	0.054
T3 (5, 7, 6)	24.6 ± 6.1	28.4 ± 6.5	31.2 ± 8.1	0.491[b]	0.097
**VAS (0-100)**					
T0 (14, 10, 8)	58.5 ± 18.6	64.3 ± 19.4	57.1 ± 25.1	0.718[a]	0.023
T1 (13, 10, 8)	79.2 ± 16.7	84.2 ± 12.5	73.3 ± 14.4	0.382[b]	0.069
T2 (13, 10, 6)	78.8 ± 20.0	84.6 ± 15.1	65.5 ± 13.6	0.152[b]	0.140
T3 (5, 7, 6)	85.0 ± 15.8	82.3 ± 10.6	79.1 ± 13.0	0.796[b]	0.032
**HOOS-Q (0-100)**					
T2 (13, 10, 6)	59.1 ± 31.3	62.5 ± 22.4	40.6 ± 8.6	0.235[a]	0.105
T3 (5, 7, 6)	85.0 ± 14.4	64.3 ± 21.0	71.9 ± 20.4	0.217[a]	0.184
**TMTa (s)**					
T0 (14, 10, 8)	98.5 (41.0-262.0)	98.0 (47.2-164.0)	75.0 (27.0-300)	0.643[c]	-
T1 (14, 10, 8)	93.7 ± 45.2	74.3 ± 20.7	95.8 ± 85.5	0.223[b]	0.102
T2 (12, 10, 6)	85.3 ± 42.5	84.5 ± 60.2	92.2 ± 79.6	0.966[b]	0.003
T3 (5, 7, 6)	53.6 ± 18.5 *	80.6 ± 34.9	84.2 ± 44.4	0.056[b]	0.338
**TMTb (s)**					
T0 (13, 10, 8)	299.0 (112.0-300.0)	231.0 (82.6-300.0)	200.0 (93.0-300.0)	0.797[c]	-
T1 (13,10, 8)	228.7 ± 86.4	194.6 ± 79.3	212.6 ± 102.8	0.418[b]	0.063
T2 (11, 10, 6)	205.7 ± 93.4	198.2 ± 86.3	195.4 ± 115.1	0.917[b]	0.007
T3 (4, 7, 6)	191.7 ± 79.5	209.2 ± 100.0	196.9 ± 105.2	0.477[b]	0.108

Measures related to fear of falling and general health in the adaptability treadmill (AT) group, conventional treadmill (CT) group and usual physical therapy (UPT) group at baseline (T0), directly after (T1), four weeks after (T2) and 12 months (T3) after the intervention. *P* values for group differences were obtained using [a] One-Way ANOVA, [b] ANCOVA with baseline performance as covariate and [c] Kruskal-Wallis test.

* Post-hoc test showed a trend towards different from usual physical therapy (UPT) ($p<0.05$)

SUBGROUP: > 1000m

	AT (n=4)	CT (n=7)	UPT (n=10)
Number of falls	1	0	9
Observation time, person-years	4.0	6.6	9.6
Fall incidence rate, falls/person-years	0.25	0.0	0.92
Number (%) of fallers	1 (25.0)	0 (0)	5 (50.0)
Incidence rate ratio (95% CI), relative to UPT	0.27(0.03– 2.9, *p*=0.281)	-	
Relative risk (95% CI), relative to UPT	0.50 (0.08- 3.0, *p*=0.580)	- (*p*=0.04)	

Abbreviations. AT: adaptability treadmill, CT: conventional treadmill, UPT: usual physical therapy, CI: confidence interval.

SUBGROUP: < 1000m

	AT (n=10)	CT (n=9)	UPT (n=6)
Number of falls	10	11	11
Observation time, person-years	9.5	8.6	5.9
Fall incidence rate, falls/person-years	1.04	1.39	1.91
Number (%) of fallers	3 (30.0)	5 (55.6)	4(66.7)
Incidence rate ratio (95% CI), relative to UPT	0.55(0.14– 2.06, *p*=0.371)	0.73 (0.19-2.75, *p*=0.640)	
Relative risk (95% CI), relative to UPT	0.45 (0.15-1.36, *p*=0.302)	0.83 (0.37-1.88, *p*=1.000)	

Abbreviations. AT: adaptability treadmill, CT: conventional treadmill, UPT: usual physical therapy, CI: confidence interval.
