# Supplementary material for: The efficacy of treadmill training with and without projected visual context for improving walking ability and reducing fall incidence and fear of falling in older adults with fall-related hip fracture: a randomized controlled trial
Source: BMC Geriatr. 2016 Dec 28;16:215. doi: 10.1186/s12877-016-0388-x (PMC5198499; doi:10.1186/s12877-016-0388-x)
Supplement: Additional file 2: — Results of the subgroup analyses related to the ability to perform Trail Making Test part B (TMTb) at baseline. (PDF 195 kb) [file 12877_2016_388_MOESM2_ESM.pdf]

SUBGROUP: ABLE TO PERFORM TMTb					
**Outcome (n: AT,CT,UPT)**	**INTERVENTION**			**Difference among groups**	
	**AT**	**CT**	**UPT**	***P* value**	**Effect size (η_p^2)**
**POMA (0-28)**					
T0 (11, 11, 15)	16.0 ± 2.5	15.6 ± 3.3	17.1 ± 3.4	0.448[a]	0.046
T1 (11, 11, 15)	19.7 ± 2.4	19.8 ± 2.2	20.1 ± 2.2	0.925[b]	0.005
T2 (9, 11, 12)	20.7 ± 1.9	21.5 ± 3.5	20.8 ± 2.9	0.478[b]	0.051
T3 (6, 8, 11)	23.2 ± 1.7	24.6 ± 2.6	24.1 ± 2.4	0.314[b]	0.104
**EMS (0-20)**					
T0 (11, 11, 15)	11.7 ± 3.7	11.4 ± 3.9	11.7 ± 3.6	0.969[a]	0.002
T1 (11, 11, 15)	15.3 ± 2.6	16.2 ± 1.7	16.1 ± 2.3	0.517[b]	0.039
T2 (9, 11, 12)	16.1 ± 2.7	16.7 ± 1.9	17.7 ± 2.3	0.192[b]	0.111
T3 (6, 8, 11)	17.8 ± 2.3	18.8 ± 1.0	18.1 ± 2.2	0.582[b]	0.059
**TUG (s)**					
T1 (11, 11, 15)	20.6 (13.5 -60.7)	18.4 (13.6-48.9)	21.5 (13.3-36.5)	0.997[c]	-
T2 (9, 11, 12)	16.2 (11.9-49.3)	16.4 (10.4-88.9)	15.6 (10.4-34.1)	0.860[c]	-
**FAC (0-5)**					
T0 (11, 11, 15)	3.0 (2.0-4.0)†	2 (2.0-3.0)*	2.0 (2.0-4.0)	0.060[c]	-
ΔT1 (11, 11, 15)	1.0 (0.0-2.0)††	2 (1.0-2.0)**	1.0 (-1.0-2.0)	**0.009**[c]	-
ΔT2 (9, 11, 12)	1.0 (0.0-2.0)††	2 (1.0-3.0)*	1.0 (0.0-3.0)	**0.009**[c]	-
ΔT3 (6, 8, 11)	1.5 (1.0-3.0)	2 (1.0-3.0)	2.0 (1.0-3.0)	0.617[c]	-
**Walking speed (m/s)**					
T1 (11, 11, 15)	0.69 ± 0.25	0.75 ± 0.28	0.69 ± 0.14	0.792[a]	0.014
T2 (9, 11, 12)	0.78 ± 0.25	0.78 ± 0.31	0.81 ± 0.22	0.894[a]	0.008
**NEADL (0-66)**					
T0 (10, 11, 15)	47.3 ± 9.3	42.3 ± 11.2*	52.1 ± 11.2	0.084[a]	0.139
T2 (9, 11, 12)	39.2 ± 16.2	42.5 ± 11.4	39.5 ± 17.5	0.265[b]	0.090
T3 (6, 8, 11)	48.3 ± 5.0	45.8 ± 4.9	46.2 ± 11.0	0.329[b]	0.100

Measures related to mobility, walking and daily functioning in the adaptability treadmill (AT) group, conventional treadmill (CT) group and usual physical therapy (UPT) group at baseline (T0), directly after (T1), four weeks after (T2) and 12 months (T3) after the intervention. *P* values for group differences were obtained using [a] One-Way ANOVA, [b] ANCOVA with baseline performance as covariate and [c]Kruskal-Wallis test.

Significant group effects are presented in bold ($p<0.05$). Post-hoc tests:

** Significantly different from usual physical therapy (UPT) ($p<0.01$)

* Trend towards different from usual physical therapy (UPT) ($p<0.05$)

†† Significantly different from conventional treadmill training (CT) ($p<0.01$)

† Trend towards different from conventional treadmill training (CT) ($p<0.05$)

SUBGROUP: UNABLE TO PERFORM TMTb					
**Outcome (n: AT,CT,UPT)**	**INTERVENTION**			**Difference among groups**	
	**AT**	**CT**	**UPT**	***P* value**	**Effect size (η_p^2)**
**POMA (0-28)**					
T0 (7, 6, 6)	14.1 ± 3.0	15.5 ± 3.4	11.8 ± 4.4	0.236 [a]	0.165
T1 (7, 6, 6)	18.7 ± 1.7*	19.3 ± 1.8*	14.8 ± 3.6	**0.035[b]**	0.361
T2 (7, 6, 5)	19.4 ± 2.9	19.2 ± 4.1	16.2 ± 3.8	0.647 [b]	0.060
T3 (1, 4, 3)	14.0 ± -	21.5 ± 4.4	19.3 ± 5.5	0.270[b]	0.480
**EMS (0-20)**					
T0 (7, 6, 6)	9.7 ± 3.4	10.5 ± 2.0	8.3 ± 3.0	0.436[a]	0.099
T1 (7, 6, 6)	15.0 ± 2.0	17.0 ± 1.3	12.8 ± 4.1	0.110[b]	0.255
T2 (7, 6, 5)	15.9 ± 2.5	15.5 ± 3.3	13.0 ± 3.9	0.474[b]	0.101
T3 (1, 4, 3)	12 ± -	16.8 ± 2.5	15.7 ± 2.1	0.371[b]	0.391
**TUG (s)**					
T1 (7, 6, 6)	26.5 (25.7-37.5)	23.9 (18.6-34.7)	42.7 (21.9-79.9)	0.182[c]	-
T2 (7, 6, 5)	25.7 (18.9-27.4)*	26.2 (17.0-37.1)	39.8 (24.1-42.2)	0.069[c]	-
**FAC (0-5)**					
T0 (7, 6, 6)	2.0 (2.0-3.0)	2.0 (2.0-4.0)	2.0 (2.0-2.0)	0.423[c]	-
ΔT1 (7, 6, 6))	2.0 (1.0-2.0)	2.0 (1.0-2.0)	1.5 (0.0-2.0)	0.416[c]	-
ΔT2 (7, 6, 5)	2.0 (1.0-2.0)	2.0 (0.0-2.0)	2.0 (0.0-2.0)	0.839[c]	-
ΔT3 (1, 4, 3)	2.0 (-)	2.0 (1.0-3.0)	2.0 (2.0-2.0)	1.000[c]	-
**Walking speed (m/s)**					
T1 (7, 6, 6)	0.56 ± 0.08	0.75 ± 0.17*	0.47 ± 0.22	**0.031[a]**	0.352
T2 (7, 6, 5)	0.67 ± 0.11*	0.61 ± 0.08	0.47 ± 0.17	0.068[a]	0.302
**NEADL (0-66)**					
T0 (7, 6, 6)	40.0 ± 10.0	42.8 ± 17.4	43.3 ± 7.7	0.869[a]	0.017
T2 (7, 6, 5)	21.3 ± 11.2	26.8 ± 17.2	18.8 ± 12.9	0.657[b]	0.058
T3 (1, 4, 3)	19.0 ± -	34.5 ± 19.2	33.0 ± 7.5	0.390[b]	0.375

Measures related to mobility, walking and daily functioning in the adaptability treadmill (AT) group, conventional treadmill (CT) group and usual physical therapy (UPT) group at baseline (T0), directly after (T1), four weeks after (T2) and 12 months (T3) after the intervention. *P* values for group differences were obtained using [a] One-Way ANOVA, [b]ANCOVA with baseline performance as covariate and [c]Kruskal-Wallis test.
Significant group effects are presented in bold (p<0.05). Post-hoc tests:
* Trend towards different from usual physical therapy (UPT) (p<0.05)

SUBGROUP: ABLE TO PERFORM TMTb					
**Outcome (n: AT, CT, UPT)**	**INTERVENTION**			**Difference among groups**	
	**AT**	**CT**	**UPT**	**P value**	**Effect size (η_p^2)**
**Obstacle effect (%)**					
T1 (11, 11, 15)	-18.1 ± 11.8	-20.5 ± 10.1	-22.0 ± 10.2	0.662[a]	0.024
T2 (9, 11, 12)	-21.0 ± 9.5	-23.7 ± 13.2	-21.5 ± 8.9	0.822[a]	0.013
**Obstacle success rate (%)**					
T1 (11, 11, 15)	100.0 (16.7-100.0)	100.0 (0.0-100.0)	83.3 (33.3-100.0)	0.639[c]	-
T2 (9, 11, 12)	100.0 (33.3-100.0)	100.0 (16.7-100.0)	100.0 (33.3-100.0)	0.868[c]	-
**DTE- walking speed (%)**					
T1 (11, 11, 15)	-27.3 ± 18.4	-36.7 ± 17.5	-32.8 ± 13.6	0.411[a]	0.051
T2 (9, 11, 12)	-29.4 ± 15.0	-29.7 ± 18.3	-18.6 ± 15.2	0.200[a]	0.105
**DTE- subtractions (%)**					
T1 (11, 11, 15)	29.0 (-33.9-208.0)	-6.2 (-62.8-55.6)	-4.8 (-43.3-48.0)	0.278[c]	-
T2 (9, 11, 12)	6.35 (-27.7-120.2)	20.0 (-16.0-58.4)	18.6 (-46.0-678.0)	0.849[c]	-

Measures related to obstacle avoidance performance and dual-task effects (DTE) in the adaptability treadmill (AT) group, conventional treadmill (CT) group and usual physical therapy (UPT) group directly after (T1) and four weeks after (T2) intervention. *P* values for group differences were obtained using [a] One-Way ANOVA and [b] Kruskal-Wallis test.

SUBGROUP: UNABLE TO PERFORM TMTb					
**Outcome (n: AT, CT, UPT)**	**INTERVENTION**			**Difference among groups**	
	**AT**	**CT**	**UPT**	**P value**	**Effect size (η_p^2)**
**Obstacle effect (%)**					
T1 (7, 6, 6)	-19.4 ± 10.3	-22.0 ± 13.9	-30.3 ± 22.0	0.463	0.092
T2 (7, 6, 5)	-23.0 ± 11.1	-25.6 ± 11.0	-22.0 ± 21.1	0.914	0.012
**Obstacle success rate (%)**					
T1 (7, 6, 6)	100.0 (50.0-100.0)*	83.3 (50.0-100.0) *	41.7 (0.0-83.3)	**0.031**	-
T2 (7, 6, 5)	100.0 (50.0-100.0)	91.7 (33.3-100.0)	83.3 (66.7-100.0)	0.832	-
**DTE- walking speed (%)**					
T1 (6, 6, 6)	-12.9 ± 15.7	-34.6 ± 20.1	-29.2 ± 20.9	0.176	0.207
T2 (6, 6, 5)	-15.2 ± 18.2	-36.8 ± 16.3	-22.8 ± 18.2	0.133	0.251
**DTE- subtractions (%)**					
T1 (5, 6, 6)	-14.3 (-100.0-11.8)	11.6 (-22.7-54.7)	-6.0 (-100.0-58.8)	0.374	-
T2 (6, 6, 5)	-43.9 (-100.0-0.0) †	-3.7 (-26.2-77.3) **	-41.1 (-100.0- -26.8)	**0.020**	-

Measures related to obstacle avoidance performance and dual-task effects (DTE) in the adaptability treadmill (AT) group, conventional treadmill (CT) group and usual physical therapy (UPT) group directly after (T1) and four weeks after (T2) intervention. *P* values for group differences were obtained using [a] One-Way ANOVA and [b] Kruskal-Wallis test. Significant group effects are presented in bold ($p<0.05$). Post-hoc tests:
** Significantly different from usual physical therapy (UPT) ($p<0.01$)
* Trend towards different from usual physical therapy (UPT) ($p<0.05$)
† Trend towards different from conventional treadmill training (CT) ($p<0.05$)

SUBGROUP: ABLE TO PERFORM TMTb					
**Outcome (n: AT, CT, UPT)**	**INTERVENTION**			**Difference among groups**	
	**AT**	**CT**	**UPT**	**P value**	**Effect size (η_p^2)**
**FES-I (20-80)**					
T0 (11, 11, 15)	42.6 ± 11.0	43.9 ± 13.6	47.4 ± 14.2	0.631[a]	0.027
T1 (11, 11, 15)	40.7 ± 16.0	39.4 ± 14.5	35.7 ± 13.1	0.268[b]	0.077
T2 (9, 11, 12)	38.1 ± 15.0	38.2 ± 16.7	30.9 ± 10.8	0.166[b]	0.120
T3 (6, 8, 11)	27.7 ± 9.3	28.6 ± 6.0	26.7 ± 7.2	0.722[b]	0.031
**VAS (0-100)**					
T0 (11, 11, 15)	62.5 ± 14.8	66.2 ± 19.4	62.8 ± 16.7	0.843[a]	0.010
T1 (10, 11, 15)	84.0 ± 19.0)	85.4 ± 10.7	73.0 ± 14.5	0.076[b]	0.148
T2 (9, 11, 12)	83.2 ± 18.7	84.2 ± 16.1	80.4 ± 12.8	0.903[b]	0.007
T3 (6, 8, 10)	82.5 ± 15.4	85.7 ± 11.6	78.5 ± 13.8	0.524[b]	0.063
**HOOS-Q (0-100)**					
T2 (9, 11, 12)	59.7 ± 30.6	59.7 ± 24.9	54.7 ± 19.8	0.861[a]	0.010
T3 (6, 8, 11)	71.9 ± 25.5	68.8 ± 20.0	77.3 ±20.2	0.688[a]	0.033
**TMTa (s)**					
T0 (11, 11, 15)	85.0 (41.0-148.0)	79.3 (47.2-122.0)	80.0 (27.0-175.0)	0.975[c]	-
T1 (11, 11, 15)	68.2 ± 26.7	70.2 ± 24.2	62.3 ± 23.2	0.413[b]	0.052
T2 (9, 11, 12)	56.6 ± 20.8	68.1 ± 27.3	54.3 ± 17.6	0.193[b]	0.111
T3 (6, 8, 11)	54.6 ± 16.9	65.7 ± 27.5	69.4 ± 26.1	0.724[b]	0.030
**TMTb (s)**					
T0 (11, 11, 15)	162.0 (100.0-299.0)	200.0 (82.6-295.5)	181.0 (80.0-290.0)	0.566[c]	-
T1 (11, 11, 15)	172.1 ± 82.5	174.4 ± 65.8	160.9 ± 71.6	0.539[b]	0.037
T2 (9, 11, 12)	132.9 ± 46.5	167.8 ± 69.6	153.3 ± 86.8	0.961[b]	0.003
T3 (6, 8, 11)	184.0 ± 70.9	181.2 ± 88.3	182.0 ± 91.6	0.389[b]	0.086

Measures related to fear of falling and general health in the adaptability treadmill (AT) group, conventional treadmill (CT) group and usual physical therapy (UPT) group at baseline (T0), directly after (T1), four weeks after (T2) and 12 months (T3) after the intervention. *P* values for group differences were obtained using [a] One-Way ANOVA, [b] ANCOVA with baseline performance as covariate and [c] Kruskal-Wallis test.

SUBGROUP: UNABLE TO PERFORM TMTb					
**Outcome (n: AT, CT, UPT)**	**INTERVENTION**			**Difference among groups**	
	**AT**	**CT**	**UPT**	**P value**	**Effect size (η_p^2)**
**FES-I (20-80)**					
T0 (7, 6, 6)	48.0 ± 15.3	46.2 ± 12.9	44.3 ± 17.4	0.912[a]	0.011
T1 (7, 6, 6)	50.6 ± 15.7 †	30.5 ± 10.8 *	50.7 ± 15.4	**0.029[b]**	0.376
T2 (7, 6, 5)	48.7 ± 11.9	37.5 ± 10.1**	59.2 ± 12.6	**0.029[b]**	0.398
T3 (1, 4, 3)	65.0 ± - **, ††	28.5 ± 6.8	30.3 ± 2.1	**0.005[b]**	0.924
**VAS (0-100)**					
T0 (7, 6, 6)	58.4 ± 23.7	70.8 ± 19.3	55.0 ± 30.4	0.519[a]	0.079
T1 (7, 6, 6)	75.7 ± 11.7	71.8 ± 20.9	84.6 ± 19.3	0.487[b]	0.091
T2 (7, 6, 5)	75.7 ± 19.0	75.0 ± 11.0	66.4 ± 21.4	0.739[b]	0.042
T3 (1, 4, 3)	50.0 ± -	77.3 ± 10.0	75.0 ± 5.0	0.121[b]	0.653
**HOOS-Q (0-100)**					
T2 (7, 6, 5)	42.9 ± 26.6	53.1 ± 21.9	40.0 ± 21.5	0.625[a]	0.061
T3 (1, 4, 3)	100.0 ± -	73.4 ± 31.6	83.3 ± 18.0	0.682[a]	0.142
**TMTa (s)**					
T0 (7, 6, 6)	114.0 (76.2-262.0)	120.5 (96.0-164.0)	143.0 (100.0-300.0)	0.257[c]	-
T1 (7, 6, 6)	127.6 ± 35.6	107.6 ± 49.2	146.4 ± 78.5	0.781[b]	0.032
T2 (6, 6, 5)	118.6 ± 28.7	152.4 ± 95.9	121.9 ± 69.6	0.404[b]	0.130
T3 (1, 4, 3)	64.4 ± -	111.4 ± 50.0	138.1 ± 41.0	0.879[b]	0.062
**TMTb (s)**					
T0 (7, 6, 6)	>300	>300)	>300	-	-
T1 (7, 6, 6)	288.6 ± 30.1	280.7 ± 47.4	300.0 ± 0.0	0.590[b]	0.064
T2 (6, 6, 5)	295.9 ± 10.1	278.8 ± 52.0	283.2 ± 37.7	0.722[b]	0.045
T3 (1, 4, 3)	300.0 ± -	246.0 ± 90.8	300.0 ± 0.0	0.589[b]	0.191

Measures related to fear of falling and general health in the adaptability treadmill (AT) group, conventional treadmill (CT) group and usual physical therapy (UPT) group at baseline (T0), directly after (T1), four weeks after (T2) and 12 months (T3) after the intervention. *P* values for group differences were obtained using [a] One-Way ANOVA, [b] ANCOVA with baseline performance as covariate and [c] Kruskal-Wallis test.
Significant group effects are presented in bold (*p*<0.05). Post-hoc tests:
** Significantly different from usual physical therapy (UPT) (*p*<0.01)
* Trend towards different from usual physical therapy (UPT) (*p*<0.05)
†† Significantly different from conventional treadmill training (CT) (*p*<0.01)
† Trend towards different from conventional treadmill training (CT) (*p*<0.05)

SUBGROUP: ABLE TO PERFORM TMTb

	AT (n=8)	CT (n=10)	UPT (n=12)
Number of falls	7	3	9
Observation time, person-years	8	9.9	11.9
Fall incidence rate, falls/person-years	0.88	0.30	0.75
Number (%) of fallers	2 (25.0)	3 (30.0)	6 (50.0)
Incidence rate ratio (95% CI), relative to UPT	1.16 (0.31– 4.41, *p*=0.824)	0.40 (0.09- 1.90, *p*=0.250)	
Relative risk (95% CI), relative to UPT	0.50 (0.013-1.89, *p*=0.373)	0.60 (0.20-1.81, *p*=0.415)	

Abbreviations. AT: adaptability treadmill, CT: conventional treadmill, UPT: usual physical therapy, CI: confidence interval

SUBGROUP: UNABLE TO PERFORM TMTb

	AT (n=5)	CT (n=6)	UPT (n=4)
Number of falls	4	8	11
Observation time, person-years	4.5	5.2	3.6
Fall incidence rate, falls/person-years	0.88	1.65	2.96
Number (%) of fallers	2 (40.0)	2 (33.3)	3 (75.0)
Incidence rate ratio (95% CI), relative to UPT	0.30 (0.05– 1.70, *p*=0.171)	0.56 (0.12-2.66, *p*=0.463)	
Relative risk (95% CI), relative to UPT	0.53 (0.16-1.80, *p*=0.524)	0.44 (0.13-1.58, *p*=0.524)	

Abbreviations. AT: adaptability treadmill, CT: conventional treadmill, UPT: usual physical therapy, CI: confidence interval.
